# Mitigating hospital-onset *Clostridioides difficile*: The impact of an optimized environmental hygiene program in eight hospitals

**DOI:** 10.1017/ice.2022.84

**Published:** 2023-03

**Authors:** Philip C. Carling, Lyndsay M. O’Hara, Anthony D. Harris, Russell Olmsted

**Affiliations:** 1 Boston University School of Medicine, Boston, Massachusetts; 2 University of Maryland, Baltimore, Maryland; 3 University of Maryland School of Medicine, Baltimore, Maryland; 4 Trinity Health, Livonia, Michigan

## Abstract

**Objective:**

To evaluate the impact of a standardized, process-validated intervention utilizing daily hospital-wide patient-zone sporicidal disinfectant cleaning on incidence density of healthcare-onset *Clostridioides difficile* infection (HO-CDI) standardized infection ratios (SIRs).

**Design:**

Multi-site, quasi-experimental study, with control hospitals and a nonequivalent dependent variable.

**Setting:**

The study was conducted across 8 acute-care hospitals in 6 states with stable endemic HO-CDI SIRs.

**Methods:**

Following an 18-month preintervention control period, each site implemented a program of daily hospital-wide sporicidal disinfectant patient zone cleaning. After a wash-in period, thoroughness of disinfection cleaning (TDC) was monitored prospectively and optimized with performance feedback utilizing a previously validated process improvement program. Mean HO-CDI SIRs were calculated by quarter for the pre- and postintervention periods for both the intervention and control hospitals. We used a difference-in-differences analysis to estimate the change in the average HO-CDI SIR and HO-CAUTI SIR for the pre- and postintervention periods.

**Results:**

Following the wash-in period, the TDC improved steadily for all sites and by 18 months was 93.6% for the group. The mean HO-CDI SIRs decreased from 1.03 to 0.6 (95% CI, 0.13–0.75; P = .009). In the adjusted difference-in-differences analysis in comparison to controls, there was a 0.55 reduction (95% CI, −0.77 to −0.32) in HO-CDI (P < .001) or a 50% relative decrease from baseline.

**Conclusions:**

This study represents the first multiple-site, quasi-experimental study with control hospitals and a nonequivalent dependent variable to evaluate a 4-component intervention on HO-CDI. Following ongoing improvement in cleaning thoroughness, there was a sustained 50% decrease in HO-CDI SIRs compared to controls.

*Clostridioides difficile* accounts for between 400,000 and 500,000 infections in the United States each year and is the leading cause of healthcare associated infections.^1,2^ The average prevalence of *C. difficile* infection (CDI) is estimated to be 13.1 per 1,000 hospitalized patients, with ∼75% being healthcare associated, making in-hospital prevention critical to decreasing the overall impact on healthcare.^3^

Three years after Bartlett et al^4^ identified *C. difficile* as the cause of antibiotic associated diarrhea in 1978, Fekety et al^5^ documented widespread healthcare environmental contamination of surfaces, both near and more distant to patients with CDI. In 1988, Katz et al^6^ described the favorable impact of a bleach-based disinfectant in decreasing environmental contamination and an associated outbreak of *C. difficile.* Subsequently, numerous quasi-experimental studies incorporating dilute bleach substitution for nonsporicidal disinfectants have reported a reduction in healthcare facility-onset CDI (HO-CDI) during outbreaks.^7^ As a result of these reports, use of a sporicidal disinfectant for daily and discharge cleaning of CDI isolation rooms has become a standard practice in acute-care hospitals during outbreaks and for endemic cases.^8^

As noted in Figure 1, many clinical research studies support the plausible benefit of optimized, daily, hospital-wide sporicidal disinfectant cleaning.^9^ However, in 2015 Kundrapu et al^10^ reported that spore shedding and near patient environmental contamination with CD spores was substantially influenced when asymptomatic *C. difficile*–colonized patients were administered antibiotics,^10^ the clinical relevance of this phenomenon has only recently been clarified. In 2016, Freedburg et al^11^ analyzed a cohort of >100,000 patients who sequentially occupied a given hospital bed. Independent of the prior room-occupant’s CDI status, administration of antibiotics to the prior bed occupant was the most significant factor associated with an increased risk of the next bed-occupant developing CDI.^11^ The same phenomenon was also reported by Dowling Root in 2021.^12^ In this study of 17,285 patient room occupancies, the risk of HO-CDI was significantly associated with prior room-occupant antibiotic usage (odds ratio, 2.37; *P* < .001). The results of these 2 large studies, which can only be explained by recipient acquisition of residual CD spores asymptomatically shed onto surfaces in the patient zone by the preceding room occupant, further support the hypothesis that daily hospital-wide sporicidal cleaning could mitigate HO-CDI.

**Fig. 1.**
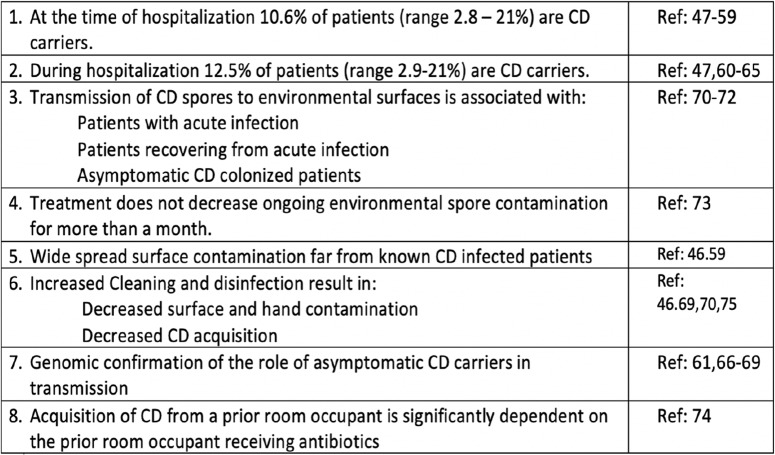
Elements of *Clostridioides* difficile environmental epidemiology.

Only when a highly effective, environmentally safe, sporicidal disinfecting chemistry^13^ which is nondamaging to surfaces^14^ became available did it become feasible to develop an assessment of such a cleaning intervention for all patient zone surfaces on the occurrence of HO-CDI. Because no study has evaluated hospital-wide sporicidal cleaning, this study was developed to analyze the impact of the concomitant implementation of daily and discharge cleaning of all inpatient rooms using a sporicidal disinfectant combined with a program to optimize thoroughness of cleaning on the HO-CDI standardized infection ratio (SIR) in an opportunity sample of 8 acute-care hospitals in the United States.

## Methods

### Study design and setting

We performed a quasi-experimental study with a control group and a nonequivalent dependent variable to evaluate the impact of objectively optimized, daily, hospital-wide, sporicidal disinfection cleaning on CDC NHSN Lab-ID reported HO-CDI standardized infection ratio (SIR).^15^ All 8 acute-care hospitals included in the intervention group were members of Trinity Health, a national multi-institutional 92-facility healthcare system. The intervention hospitals ranged in size from a 532-bed tertiary-care hospital to a 44-bed regional critical-access hospital (mean, 257 beds) located in 6 geographically diverse states. Furthermore, 9 randomly selected Trinity Health hospitals that had not enrolled in the standardized system environmental services (EVS) program (intervention) served as controls. The control hospitals ranged from 552 to 44 beds (mean, 266 beds). Prior to the intervention period, all hospitals were using a dilute bleach-based protocol for daily and discharge room cleaning of rooms occupied by patients with CDI, and none had implemented disinfection cleaning thoroughness monitoring and optimization per CDC level II recommendations.^16^ Thoroughness of disinfection cleaning (TDC) is defined as the proportion of actually cleaned objects in a room compared to the number of objects recommended to be cleaned by policy, and expressed as a percentage. TDC was monitored in an identical fashion by all study sites, as previously described, using a standardized fluorescent marking system (DAZO, Ecolab, St. Paul, MN) specifically developed not to be easily visible to the EVS staff.^17,18^ Outcome data were collated centrally but were not shared between the sites and were not used for educational purposes during the study.

### The Intervention

Following a 30- to 60-day period of covert evaluation of cleaning thoroughness, all EVS technicians and managers participated in a structured and standardized educational program consisting of both classroom and hands-on training as part of Trinity Health’s C.L.E.A.N. Suite EVS Program, which that was operated from the system’s headquarters. Prior to and during the training they continued to perform traditional disinfection cleaning: general patient zone daily and discharge cleaning of surfaces with a quarternary ammonium disinfectant substituting dilute bleach for cleaning rooms use to isolate patients with CDI. Each site independently implemented the intervention protocol at various time points during 2017 and was evaluated over 18 months. During the intervention, daily hospital-wide, patient-zone, surface-disinfection cleaning was implemented using a 1-step sporicidal disinfectant cleaner. This hydrogen peroxide-peroxyacetic acid-based, disinfectant (OxyCide Daily Disinfectant Cleaner, Ecolab, St. Paul, MN) has a 5-minute contact time for efficacy against *C. difficile* spores and a 3-minute contact time for most other bacteria and viruses.^13^ Concomitantly, a structured performance monitoring and feedback program using the fluorescent marker system, compliant with CDC guidance level II recommendations, was used to optimize cleaning practice.

### Outcomes

The primary outcome, NSHN Lab-ID–reported HO-CDI (SIR) results were collected monthly and were collated centrally by quarter for 18 months prior to beginning the program and for 18 months following the wash-in period. Following the 3-month wash-in period, disinfection cleaning monitoring results were collected on an ongoing basis to provide objective analysis cleaning practice to optimize process improvement through direct feedback and education.^17,19^ During the 39 months of the intervention all cleaning evaluations were done by infection preventionists and/or trained individuals not directly involved in environmental cleaning activities.

### Other variables of interest

Because the HO-CDI SIR may be affected by the severity of hospitalized patient illness and the duration of hospitalization, we explored the average length of stay and Medicare case-mix index^20^ for each hospital to assess trends that might impact HO-CDI for both intervention and control sites. NHSN reported HO–catheter-associated urinary tract infections (HO-CAUTI) SIRs were evaluated as a nonequivalent dependent variable (or “removed treatment design”) that should be affected by similar hospital-level factors as HO CDI but not by the intervention studied.^21,22^ We also evaluated 6 other variables that could have impacted HO-CDI SIRs using a standardized questionnaire including changes in transmission-based precautions, specimen collection stewardship, the *C. difficile* laboratory testing method used, new antimicrobial stewardship initiatives and proton-pump inhibitor use.

### Statistical analysis

The mean and interquartile range of TDC scores were calculated by quarter for the pre- and postintervention periods for the 8 intervention hospitals. Similarly, mean and interquartile range of HO-CDI standardized infection ratios were calculated by quarter for the pre- and postintervention periods for both the intervention and the control hospitals. We used a difference-in-differences analysis to estimate the change in the average HO-CDI SIR and HO-CAUTI SIR for the pre- and postintervention periods.^23^ We fit linear mixed-effects regression models for each outcome as a function of intervention or control group status, the pre- or postintervention period, and their interaction. The model also included a hospital indicator as a random intercept and a time trend indicator. The interaction term is the difference-in-differences estimate of the association between the intervention and HO-CDI or HO-CAUTI.^24^

## Results

### Thoroughness of cleaning

Each hospital evaluated their preintervention disinfection cleaning over 1–2 months between the last quarter of 2016 (one site) and quarter 3 of 2017. Prior to implementation of the intervention, covert evaluation of cleaning thoroughness showed that 59% of policy defined patient zone surfaces were being disinfection cleaned by environmental services personnel (range, 21%–72% (Table 1). Following the wash-in period, the TDC improved for all sites to 86% during (95% CI, 39%–13%; *P* = .0007) (Fig. 2A). Subsequently cleaning thoroughness continued to improve over the next 5 quarters and at 18 months was 93.6% for the group (range, 91%–96%; 95% CI, 45%–24%; *P* < .0001).

**Table 1.** Intervention and Control Hospital Results.

										Mean	Delta
Study Hospital Bed Size		532	451	357	204	192	176	98	44	257	
Pre-Intervention											
	Baseline TDC	54	72	27	58	68	70	62	61	59	
	HO-CDI SIR	1.35	0.9	1	1.13	1.17	1.42	0.49	0.75	1.03	
	ALOS	5.9	4.6	5	3.2	4.4	4.2	3.4	4.6	4.4	
	MCCMI	2.3	1.9	1.9	1.4	1.8	1.6	1.6	1.6	1.76	
Post-Intervention											
	TDC Q 1	94	88	92	93	81	88	88	65	86	+ 46%
	TDC by Q 6	94	95	91	92	96	92	94	95	93.6	+ 59%
	HO-CDI SIR Q1	0.6	0.5	0.7	0	0.9	0.7	0.6	0.6	0.6	− 42%
	HO-CDI Q4-Q6	0.38	0.44	0.39	0.07	0.27	0.1	0.41	0.35	0.30	− 69%
	ALOS	6	4.4	4.8	3.2	4.9	4.4	3.5	2.5	4.2	− 5%
	MCCMI	2.3	1.9	1.9	1.5	1.9	1.6	1.6	1.5	1.78	+ 1%
Control Hospital Bed Size		552	441	317	304	235	234	191	73	44	266
	HO-CDI SIR 2017	0.8	1	0.5	0.9	0.7	0.4	0.7	1.3	0.5	0.76
	HO-CDI SIR 2018	0.8	0.9	0.6	1.4	0.7	0.4	0.4	0.6	0.6	0.71
	ALOS 2018	4.6	5.5	5.3	4.7	4.5	4.5	5.9	4.5	2.8	4.7
	MCCMI 2018	1.9	1.8	1.5	1.5	1.8	1.9	1.6	1.6	1.5	1.7

Note: Delta, proportional difference between preintervention and postintervention mean values; TDC, thoroughness of disinfection cleaning; HO-CDI SIR, healthcare-onset *C. difiicile* standardized infection rate; ALOS, average length of stay; MCCMI, Medicare case-mix index; Q1, quarter 1; Q6, quarter 6.

**Fig. 2A:**
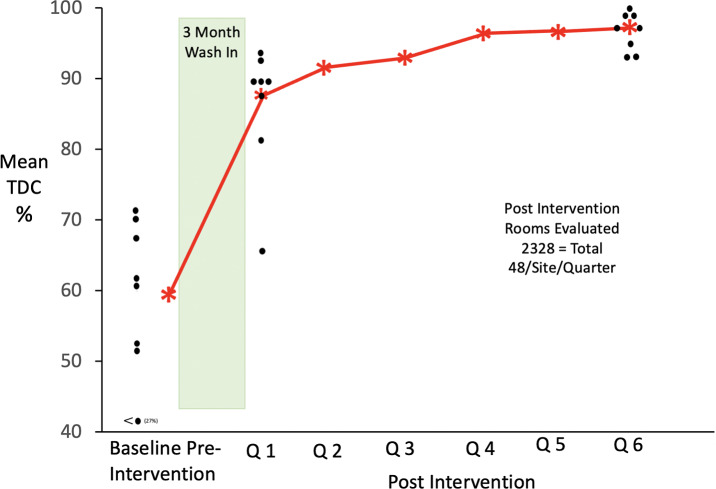
. Thoroughness of cleaning in 8 intervention hospitals.

### Hospital-onset *C. difficile* infection

As noted in Figure 2B, mean group HO-CDI SIRs clustered from 0.49 to 1.42 above and below a mean of 1.03 during the 18 months prior to project implementation. In quarter 1, following the wash-in period, all sites documented a decrease in HO-CDI to a mean SIR of 0.6 (95% CI, 0.13–0.75, *P* = .009). Over the next 5 quarters, the HO-CDI SIR continued to decrease and stabilized during the last three quarters evaluated to a mean SIR of 0.6 (95% CI, 0.13–0.75; *P* = .009). In the adjusted difference-in-differences analysis (Fig. 3A), the intervention was associated with a 0.55 reduction (95% CI, −0.77 to −0.32) in HO-CDI (*P* < .001; or a 50% relative decrease from a baseline SIR of 1.03).

**Fig. 2B.**
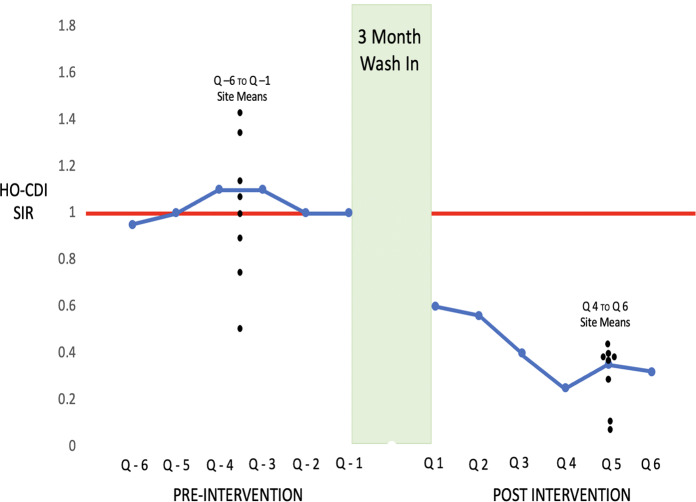
Endemic HO-SIRs in 8 intervention hospitals.

**Fig. 3.**
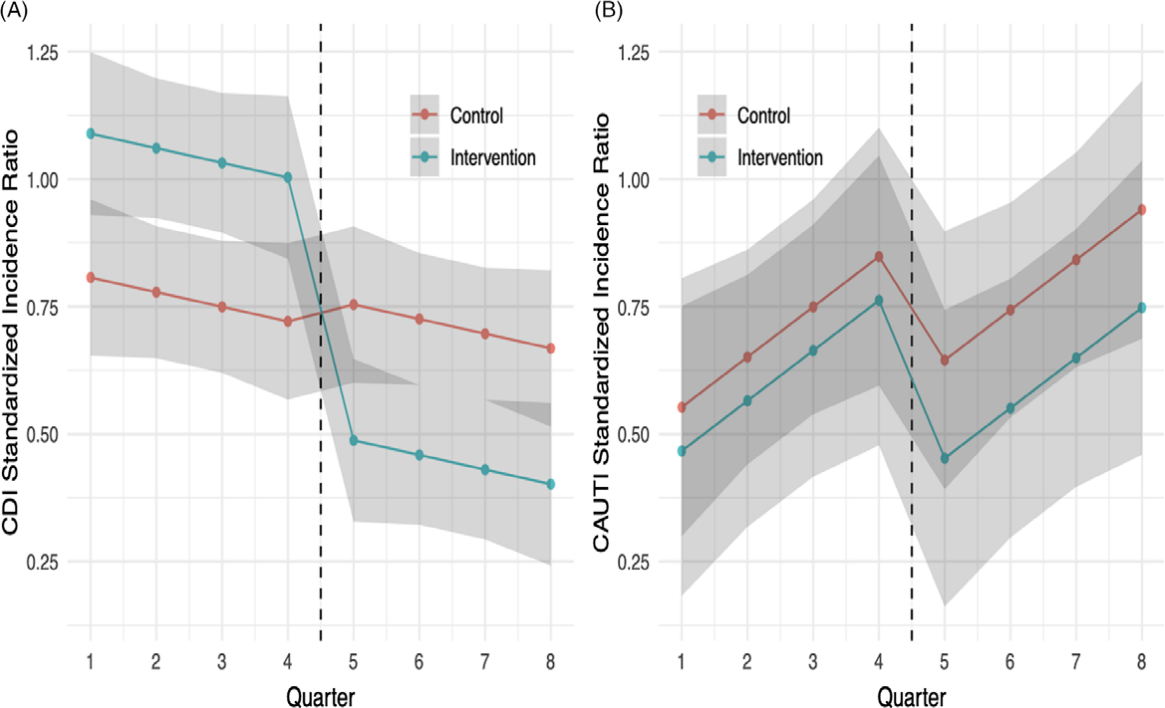
Difference-in-difference analysis of (A) hospital-onset *C. difficile* infection (HO-CDI) and (B) hospital-onset catheter-associated urinary tract infection (HO-CAUTI).

**Fig. 4.**
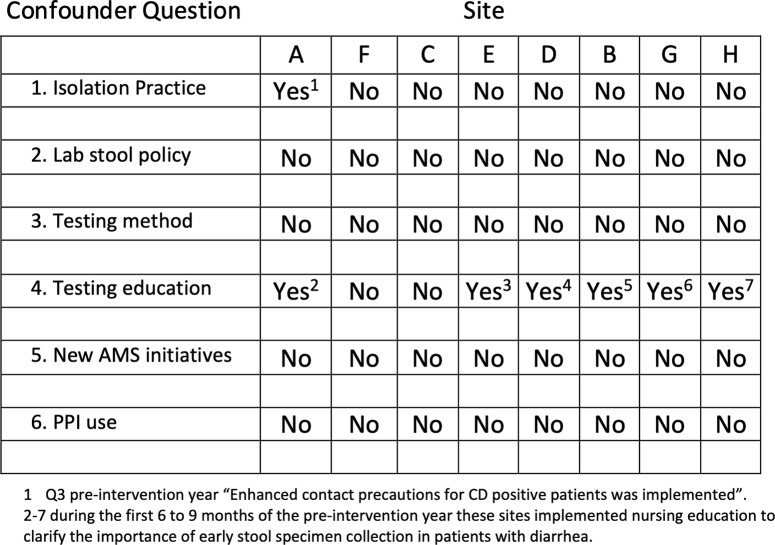
Causal variables possibly effecting outcomes.

### Hospital-onset catheter-associated urinary tract infection

With respect to the nonequivalent dependent variable analysis and as noted in Figure 3B, the intervention was not associated with a concomitant trend in HO-CAUTI SIRs (0.11; 95% CI, −0.50 to 0.29; *P* = .60).

### Other potential confounders

With the exception of 1 site that restructured transmission-based precautions for known CDI patients in quarter 3 of the preintervention year, no other site modified isolation policies or procedures related to CDI (Fig. 4). None of the sites modified previously implemented stool sample stewardship procedures. All sites had implemented polymerase chain reaction (PCR)-based testing several years previously. Six sites implemented nursing education activities to clarify the importance of early stool specimen collection in all patients with diarrhea during the preintervention period. All sites had antimicrobial stewardship programs in place for several years prior to 2017, which remained unchanged during the intervention period. In addition, no changes in proton pump inhibitor use were noted. Acuity of care as measured by the Medicare case-mix index was 1.76 for the intervention group during the preintervention period and was unchanged during the postintervention year (mean, 1.78) (Table 1). Similarly, the average length of stay remained stable. (2017 mean, – 4.4 days; 2018 mean, 4.2 days) during the pre- and postintervention periods.

## Discussion

This study represents the first multisite, quasi-experimental study with control hospitals and a nonequivalent dependent variable to evaluate a 4-component intervention on HO-CDI. After an 18-month preintervention period and immediately following a 3-month wash-in period, there was a 46% improvement in cleaning from 59% to 86% immediately following the wash-in period with 5 sites at ≥88% (*P* = .0009) (Fig. 2A) Over the next 15 months, with ongoing objectively documented performance feedback to the environmental services staff, disinfection cleaning improved steadily for all intervention sites, ultimately reaching 93.6% (range, 91%–96% by quarter 6; *P* ≤ .0001) Concomitantly, NHSN reported HO-CDI decreased 52% in quarter 1 in the intervention hospitals (*P* = .0009) (Fig. 2B). Over the next 5 quarters, HO-CDI SIRs continued to decrease, stabilizing during the last 9 months of the study and falling to a mean SIR of 0.30 (range, 0.1–.59; *P* = .0009), by 18 months after the intervention. Although sites B and A, with the highest SIRs prior to implementing the program, showed large decreases in HO-CDI (93% and 84%) could have biased the overall results of this relatively small study, the 3 sites with preintervention SIRs <1.0 improved by 40%, 40%, and 44%, along with the sustained responses noted by all sites. This finding suggests that even sites with low endemic rates of HO-CDI could benefit from a program that includes hospital-wide daily sporicidal disinfection cleaning, education, monitoring, and feedback to mitigate endemic HO-CDI.

Our findings suggest that the intervention program was associated with the observed decrease in HO-CDI for the intervention sites. This conclusion is supported by the following findings: The intervention site patient acuity was stable over 39 months. The endemic HO-CDI SIRs before the intervention were stable. There was an abrupt, concomitant increase in cleaning thoroughness and decrease in CDI in the first quarter of the intervention, which was sustained over 2 years. We also utilized a “removed treatment design”^21,22^ and showed that the nonequivalent dependent variable of CAUTI SIR did not change during the intervention period in intervention hospitals. Lastly the other hospital-level factors that could have driven lower *C. difficile* rates showed no identifiable changes between the preintervention and postintervention periods.

The potential value of broadly implemented sporicidal cleaning to mitigate HO-CDI was discussed by Freedberg et al^11^ in the previously noted report. In reviewing their finding that antibiotic administration to prior room occupant was the most important risk factor for CDI development in the subsequent room occupants, it was suggested that there could be a benefit from “focusing cleaning protocols on the rooms of patients who have a risk factor for CDI (eg, recipt of antibiotics)”^11p.E6^ Although such a program could be implemented, the fact that most hospitalized patients receive antibiotics along with the frequent occurrence of patients occupying more than a single room during their hospitalization would make such a program logistically challenging.

This study has several limitations. Unmeasured factors might have limited the generalizability of our findings. However, the fact that there was a wide range in the location of the intervention sites, their size and patient acuity, as well as the fact that the preintervention SIR for the group was at the national norm (SIR, 1.0) for NHSN-reported HO-CDI SIRs in 2017 supports the plausible generalization of our findings. Some hospitals might not be able to achieve high levels of disinfection cleaning, thereby limiting the impact of hospital-wide sporicidal cleaning. However, 77 hospitals that implementing CDC guidance level II disinfection cleaning monitoring identical to that described in this report all documented sustained improvement in TDC (baseline vs improved) from 48% to 73% in 36 hospitals (2008),^17^ 24% to >90% in 12 hospitals (2010),^25^ 61% to 89% in 22 hospitals (2017),^26^ and 63% to 82% in 9 hospitals (2017).^27^ Limitations of the study design precluded evaluation of the relative impact of each of the individual components of the program. However, previous studies have reported the following findings: (1) that environmental spore contamination decreased only after disinfection cleaning had been optimized in 9 intervention hospitals compared to controls^26^; (2) that a good level of cleaning thoroughness was ineffective in decreasing environmental spore contamination until daily cleaning had been implemented^27^; (3) that a high level of disinfection cleaning had no impact on HO-CDI until hospital wide sporicidal cleaning was implemented in a single site study^28^; and (4) that daily sporicidal cleaning failed to impact HO-CDI over 2 years until a program to optimize TCD was implemented.^29^ These findings suggest that each of the components of our intervention are critical to achieving the impact on HO-CDI presented in this report.

A randomized controlled trial could, in theory, further clarify and quantify the results of this intervention, but such an undertaking would require some sites to defer implementing potentially effective design elements of the intervention. The difference-in-differences analysis used in this study is arguably the “next best” approach when randomization is not appropriate or feasible. The strength of the difference-in-differences approach stems from the fact that it uses a comparison group that is experiencing the same trends but is not exposed to the intervention.^23^ Since it compares outcomes between the intervention group and the control group without the exposure (preimplementation) and the intervention group to itself with the exposure (postimplementation), the investigator is able to subtract the background changes in outcomes.^22^ Given the challenges of a randomized trial to answer our question of interest, we note an agent-based modeling study by Barker et al (2020) that evaluated the impact of multiple single and bundled interventions on HO-CDI prevention and found that the single most clinically effective as well as cost-effective intervention was daily sporicidal disinfection cleaning of all patient zone surfaces.^30^ Furthermore, quantative input analysis of the model found that “enhanced level” (80% TDC) provided benefits (as seen in this study), but that there was only a limited additional incremental benefit from increasing modeling parameters of thoroughness of cleaning from an “enhanced level” (80% TDC) to an “ideal level” (94% TDC). Thus, daily patient zone sporicidal cleaning could realize a substantial impact on *C. difficile* transmission at TDC levels lower than those achieved by this group of hospitals.

Notably, the design of our intervention program also is in line with several elements of the CDC October 2020 guidance *Core Components of Environmental Cleaning and Disinfection*,^31^ including the following: ongoing integration of environmental services and infection prevention activities (component 1); use of standardized cleaning and disinfection chemistry products tailored to the setting (component 3); optimized, setting-specific cleaning and disinfection protocols (component 4); and the use of objective ongoing programmatic performance monitoring to optimize disinfection cleaning (components 2, 5, and 6).

This study provides support for the clinical benefit of an integrated 4-component, intervention, not bundled with other transmission mitigating activities, focusing solely on environmental hygiene practice that includes daily, hospital-wide sporicidal disinfectant cleaning with objectively optimized thoroughness of cleaning to reduce endemic HO-CDI. Although optimizing other bundled interventions might lead to additional mitigation of HO-CDI beyond the impact of the intervention described in this report, Baker et al^30^ found relatively little cost-benefit of such measures once a hospital implemented daily sporicidal cleaning and achieved a TDC of 80 to 94%.

Notably, multiple recent studies provide strong support for the likely benefit of the intervention described in this report in mitigating transmission of a wide range of healthcare-associated pathogens, including VRE,^32,33^ MRSA,^33,34^ MDROs,^35–39^ CRE capable of plasmid-mediated carbenapenemase gene transfer^36^ and organisms such as norovirus and *Candida auris.* Along with facilitating environmental control of *C. difficile* transmission such a broadly horizontal approach to healthcare-associated pathogen transmission supports the guidance provided in the Commentary, “Approaches for preventing healthcare-associated infections: Go Long or Go Wide?” by Septimus et al^40^ as well as the recent CDC guidance, *Core Elements of Hospital Environmental Hygiene*.^30^
